# Bis(μ-*N*-benzyl-*N*-tetra­decyl­dithio­carbamato-κ^2^ *S*:*S*′)bis­[(*N*-benzyl-*N*-tetra­decyl­dithio­carbamato-κ^2^ *S*,*S*′)zinc(II)]

**DOI:** 10.1107/S1600536809011155

**Published:** 2009-03-31

**Authors:** Chun-Man Jia, Wen-Bing Yuan, Qiang Lin, Qi Zhang, Jie Pei

**Affiliations:** a Hainan Provincial Key Laboratory of Fine Chemicals, Hainan University, Renmin Avenue 58, Haikou 570228, People’s Republic of China

## Abstract

In the title compound, [Zn_2_(C_22_H_36_NS_2_)_4_], two bidentate dithio­carbamate groups chelate directly to the Zn^II^ atoms, whereas the two remaining dithio­carbamate ligands bridge the Zn atoms *via* a crystallographic inversion centre. The Zn atoms show a strongly distorted tetra­hedral geometry. Adding the long S⋯S distance with the inversion centre being in the middle, the resulting five-coordinate geometry around the Zn atoms can be considered to be between distorted recta­ngular pyramidal and trigonal bipyramidal, with a calculated τ value of 0.31. In this dimer complex, two inversion-related tetra­decyl carbon chains exhibit all-*trans* conformations, and the other two chains show a *cis* conformation at the end of the chains.

## Related literature

For related centrosymmetric dimeric Zn^II^ structures, see: Baba, Farina, Othman *et al.* (2001 ▶); Baba, Farina, Kassim *et al.* (2001 ▶); Shaheen *et al.* (2006 ▶). For an analysis of five-coordinate metal atoms in the crystalline state, see: Addison *et al.* (1984 ▶).
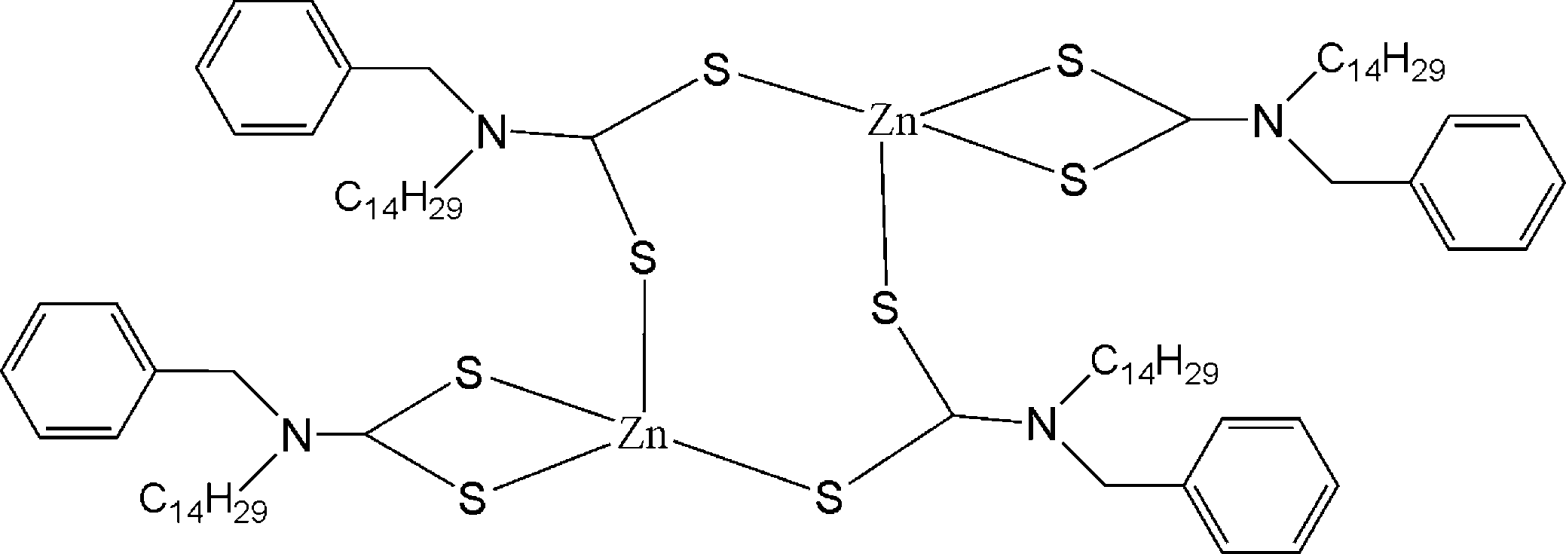


## Experimental

### 

#### Crystal data

[Zn_2_(C_22_H_36_NS_2_)_4_]
*M* *_r_* = 1645.29
Triclinic, 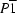

*a* = 11.007 (1) Å
*b* = 11.640 (1) Å
*c* = 18.818 (2) Å
α = 85.645 (4)°
β = 76.913 (4)°
γ = 73.263 (4)°
*V* = 2248.7 (4) Å^3^
*Z* = 1
Mo *K*α radiation
μ = 0.76 mm^−1^
*T* = 153 K
0.43 × 0.10 × 0.10 mm

#### Data collection

Rigaku SPIDER diffractometer
Absorption correction: empirical (using intensity measurements) (*ABSCOR*; Higashi, 1995 ▶) *T*_min_ = 0.734, *T*_max_ = 0.928
15746 measured reflections
7979 independent reflections
7057 reflections with *I* > 2σ(*I*)
*R*_int_ = 0.024

#### Refinement

*R*[*F* ^2^ > 2σ(*F* ^2^)] = 0.037
*wR*(*F* ^2^) = 0.087
*S* = 1.01
7979 reflections
462 parameters
H-atom parameters constrained
Δρ_max_ = 0.37 e Å^−3^
Δρ_min_ = −0.25 e Å^−3^

### 

Data collection: *RAPID-AUTO* (Rigaku, 2004 ▶); cell refinement: *RAPID-AUTO*; data reduction: *RAPID-AUTO*; program(s) used to solve structure: *SHELXS97* (Sheldrick, 2008 ▶); program(s) used to refine structure: *SHELXL97* (Sheldrick, 2008 ▶); molecular graphics: *SHELXTL* (Sheldrick, 2008 ▶); software used to prepare material for publication: *SHELXTL* and *PLATON* (Spek, 2009 ▶).

## Supplementary Material

Crystal structure: contains datablocks I, global. DOI: 10.1107/S1600536809011155/si2160sup1.cif

Structure factors: contains datablocks I. DOI: 10.1107/S1600536809011155/si2160Isup2.hkl

Additional supplementary materials:  crystallographic information; 3D view; checkCIF report

**Fig. 1.**
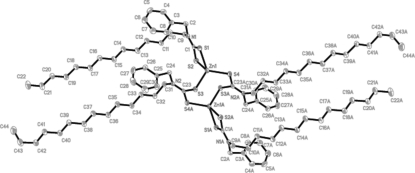
A perspective view of the dimer complex of (I). Displacement ellipsoids are drawn at the 50% probability level. Symmetry code for the atoms labelled with an A: (2 - x, -y, 1 - z).

Enhanced figure: interactive version of Fig. 1

## Figures and Tables

**Table:** 

Zn1—S4	2.3396 (6)
Zn1—S2	2.3398 (6)
Zn1—S3	2.3711 (6)
Zn1—S1	2.4420 (6)
Zn1—S3^i^	2.8879 (6)

**Table:** 

S4—Zn1—S2	136.18 (2)
S1—Zn1—S3^i^	154.92 (2)

Symmetry code: (i) 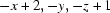.

## supplementary crystallographic information

### Comment

Some crystal structures of centrosymmetric dimeric zinc^II^–dithiocarbamate complexes have been reported, and this family compounds involve the similar ligands such as ethylisopropyldithiocarbamate (Baba, Farina, Othman *et al.*, 2001), ethylbutyldithiocarbamate (Baba, Farina, Kassim *et al.*, 2001) and piperidine-1-dithiocarbamate (Shaheen *et al.*,2006).

In the title complex (I), representing another member of dimeric dithiocarbamate complexes, two inversion related tetradecyl carbon chains exhibit all *trans*-conformations, and the other two chains show a *cis*-conformation at the end of the chains. The Zn–S bond lengths are within the sum of the covalent radii of 2.47 Å (S = 1.02 Å, Zn = 1.45 Å) (Table 1), and they agree with the values found in the literatures (Baba, Farina, Othman *et al.*, 2001; 2001b; Shaheen *et al.*, 2006). However, two of the six tetrahedral angles [S2–Zn1–S1=75.71°(2) and S4–Zn1–S2=136.18°(2)] differ greatly from the ideal value, 109.5°. Consequently, the longer distance of 2.8879 (6) Å for Zn1–S3A may be considered, which expands the view of a strongly distorted tetrahedral ZnS_4_ evironment: if the symmetry related atom S3A (symmetry code: 2-x, -y, 1-z) is added to the Zn environment, a rectangular pyramidal or a trigonal bipyramidal geometry can be calculated by using the formula τ = (β - α)/60, which is applicable to five-co-ordinate structures within the structural continuum between trigonal bipyramidal and rectangular pyramidal (Addison *et al.*, 1984). In this structure, the "rectangular" unit consists of S1, S2 S4, S3A, and S3 is considered as the axial atom. The largest angles within the four atoms S1-S3A are β = 154.92 (2)° for S1–Zn1–S3A and α = 136.18 (2)° for S2–Zn1–S4. As a result, τ is (154.92-136.18)/60 = 0.31, indicating a 69% rectangular pyramidal geometry.

### Experimental

White crystals of (I) were obtained by slow evaporation of a solution in dichloromethane (10 ml) of benzyl(tetradecyl)carbamatodithioic acid (0.076 g, 0.2 mmol) and Zn(OAc)_2_ (0.022 g, 0.1 mmol).

### Refinement

H atoms were positioned geometrically (C–H = 0.95–0.99 Å) and allowed to ride on their parent atoms with *U*_iso_(H) = 1.2 *U*_eq_(C).

### Crystal data

**Table:**

[Zn_2_(C_22_H_36_NS_2_)_4_]	*Z* = 1
*M**_r_* = 1645.29	*F*(000) = 888
Triclinic, *P*1	*D*_x_ = 1.215 Mg m^−^^3^
Hall symbol: -P 1	Mo *K*α radiation, λ = 0.71073 Å
*a* = 11.007 (1) Å	Cell parameters from 6628 reflections
*b* = 11.640 (1) Å	θ = 3.0–27.5°
*c* = 18.818 (2) Å	µ = 0.76 mm^−^^1^
α = 85.645 (4)°	*T* = 153 K
β = 76.913 (4)°	Claviform, white
γ = 73.263 (4)°	0.43 × 0.10 × 0.10 mm
*V* = 2248.7 (4) Å^3^	

### Data collection

**Table:**

Rigaku SPIDER diffractometer	7979 independent reflections
Radiation source: Rotating Anode	7057 reflections with *I* > 2σ(*I*)
graphite	*R*_int_ = 0.024
Detector resolution: 28.5714 pixels mm^-1^	θ_max_ = 25.3°, θ_min_ = 3.0°
ω scans	*h* = −13→13
Absorption correction: empirical (using intensity measurements) (*ABSCOR*; Higashi, 1995)	*k* = −13→13
*T*_min_ = 0.734, *T*_max_ = 0.928	*l* = −22→22
15746 measured reflections	

### Refinement

**Table:**

Refinement on *F*^2^	Primary atom site location: structure-invariant direct methods
Least-squares matrix: full	Secondary atom site location: difference Fourier map
*R*[*F*^2^ > 2σ(*F*^2^)] = 0.037	Hydrogen site location: inferred from neighbouring sites
*wR*(*F*^2^) = 0.087	H-atom parameters constrained
*S* = 1.01	*w* = 1/[σ^2^(*F*_o_^2^) + (0.0466*P*)^2^ + 0.22*P*] where *P* = (*F*_o_^2^ + 2*F*_c_^2^)/3
7979 reflections	(Δ/σ)_max_ = 0.001
462 parameters	Δρ_max_ = 0.37 e Å^−^^3^
0 restraints	Δρ_min_ = −0.25 e Å^−^^3^

### Special details

**Table:**

Geometry. All e.s.d.'s (except the e.s.d. in the dihedral angle between two l.s. planes) are estimated using the full covariance matrix. The cell e.s.d.'s are taken into account individually in the estimation of e.s.d.'s in distances, angles and torsion angles; correlations between e.s.d.'s in cell parameters are only used when they are defined by crystal symmetry. An approximate (isotropic) treatment of cell e.s.d.'s is used for estimating e.s.d.'s involving l.s. planes.
Refinement. Refinement of *F*^2^ against ALL reflections. The weighted *R*-factor *wR* and goodness of fit *S* are based on *F*^2^, conventional *R*-factors *R* are based on *F*, with *F* set to zero for negative *F*^2^. The threshold expression of *F*^2^ > σ(*F*^2^) is used only for calculating *R*-factors(gt) *etc*. and is not relevant to the choice of reflections for refinement. *R*-factors based on *F*^2^ are statistically about twice as large as those based on *F*, and *R*- factors based on ALL data will be even larger.

### Fractional atomic coordinates and isotropic or equivalent isotropic displacement parameters (Å^2^)

**Table:**

	*x*	*y*	*z*	*U*_iso_*/*U*_eq_	
Zn1	0.83591 (2)	0.04989 (2)	0.478389 (13)	0.02398 (8)	
S1	0.62087 (5)	0.16828 (5)	0.46274 (3)	0.02492 (13)	
S2	0.69528 (5)	0.01501 (5)	0.58593 (3)	0.02590 (13)	
S3	0.97178 (5)	0.16703 (4)	0.49421 (3)	0.02298 (12)	
S4	0.94700 (5)	−0.05478 (5)	0.37081 (3)	0.02584 (13)	
N1	0.44801 (16)	0.12946 (15)	0.58110 (9)	0.0223 (4)	
N2	0.83787 (15)	0.23154 (14)	0.63025 (9)	0.0220 (4)	
C1	0.57256 (19)	0.10751 (17)	0.54731 (11)	0.0219 (4)	
C2	0.3428 (2)	0.20710 (19)	0.54799 (12)	0.0272 (5)	
H2A	0.2691	0.1714	0.5580	0.033*	
H2B	0.3746	0.2089	0.4944	0.033*	
C3	0.29449 (19)	0.33458 (19)	0.57567 (11)	0.0263 (5)	
C4	0.1671 (2)	0.3788 (2)	0.61380 (13)	0.0347 (5)	
H4	0.1105	0.3287	0.6223	0.042*	
C5	0.1217 (2)	0.4952 (2)	0.63952 (14)	0.0421 (6)	
H5	0.0347	0.5240	0.6660	0.050*	
C6	0.2020 (2)	0.5698 (2)	0.62687 (14)	0.0404 (6)	
H6	0.1705	0.6499	0.6440	0.049*	
C7	0.3282 (2)	0.5264 (2)	0.58913 (13)	0.0369 (6)	
H7	0.3844	0.5769	0.5806	0.044*	
C8	0.3742 (2)	0.4103 (2)	0.56352 (12)	0.0300 (5)	
H8	0.4615	0.3820	0.5373	0.036*	
C9	0.4060 (2)	0.06529 (19)	0.64844 (11)	0.0266 (5)	
H9A	0.4836	0.0071	0.6611	0.032*	
H9B	0.3490	0.0190	0.6386	0.032*	
C10	0.3337 (2)	0.1449 (2)	0.71421 (11)	0.0278 (5)	
H10A	0.2503	0.1959	0.7040	0.033*	
H10B	0.3132	0.0931	0.7568	0.033*	
C11	0.4075 (2)	0.22489 (19)	0.73399 (11)	0.0279 (5)	
H11A	0.4242	0.2798	0.6925	0.033*	
H11B	0.4926	0.1745	0.7422	0.033*	
C12	0.3352 (2)	0.2987 (2)	0.80179 (12)	0.0332 (5)	
H12A	0.2468	0.3428	0.7955	0.040*	
H12B	0.3262	0.2437	0.8442	0.040*	
C13	0.4018 (2)	0.3877 (2)	0.81796 (12)	0.0340 (5)	
H13A	0.4916	0.3435	0.8218	0.041*	
H13B	0.4078	0.4441	0.7760	0.041*	
C14	0.3365 (2)	0.4600 (2)	0.88661 (12)	0.0342 (5)	
H14A	0.3318	0.4043	0.9289	0.041*	
H14B	0.2465	0.5042	0.8833	0.041*	
C15	0.4060 (2)	0.5490 (2)	0.89997 (12)	0.0341 (5)	
H15A	0.4112	0.6042	0.8574	0.041*	
H15B	0.4959	0.5046	0.9034	0.041*	
C16	0.3417 (2)	0.6231 (2)	0.96837 (13)	0.0341 (5)	
H16A	0.2511	0.6661	0.9656	0.041*	
H16B	0.3387	0.5682	1.0112	0.041*	
C17	0.4102 (2)	0.7137 (2)	0.97988 (12)	0.0340 (5)	
H17A	0.4108	0.7700	0.9377	0.041*	
H17B	0.5016	0.6709	0.9810	0.041*	
C18	0.3486 (2)	0.7857 (2)	1.04965 (12)	0.0336 (5)	
H18A	0.2567	0.8274	1.0490	0.040*	
H18B	0.3495	0.7296	1.0919	0.040*	
C19	0.4158 (2)	0.8777 (2)	1.06021 (12)	0.0326 (5)	
H19A	0.4135	0.9346	1.0183	0.039*	
H19B	0.5081	0.8362	1.0599	0.039*	
C20	0.3563 (2)	0.9484 (2)	1.13037 (12)	0.0315 (5)	
H20A	0.2640	0.9899	1.1307	0.038*	
H20B	0.3588	0.8916	1.1723	0.038*	
C21	0.4238 (2)	1.0405 (2)	1.14052 (13)	0.0410 (6)	
H21A	0.4230	1.0964	1.0981	0.049*	
H21B	0.5157	0.9989	1.1413	0.049*	
C22	0.3622 (3)	1.1126 (2)	1.20980 (14)	0.0517 (7)	
H22A	0.2712	1.1543	1.2095	0.062*	
H22B	0.4091	1.1715	1.2124	0.062*	
H22C	0.3664	1.0585	1.2523	0.062*	
C23	0.94184 (19)	0.15950 (17)	0.58946 (11)	0.0211 (4)	
C24	0.73607 (19)	0.31852 (18)	0.59877 (12)	0.0262 (5)	
H24A	0.6508	0.3052	0.6215	0.031*	
H24B	0.7529	0.3036	0.5459	0.031*	
C25	0.72970 (19)	0.44683 (18)	0.60955 (11)	0.0221 (4)	
C26	0.6207 (2)	0.5230 (2)	0.65140 (12)	0.0317 (5)	
H26	0.5483	0.4944	0.6737	0.038*	
C27	0.6166 (2)	0.6411 (2)	0.66099 (14)	0.0427 (6)	
H27	0.5416	0.6928	0.6901	0.051*	
C28	0.7200 (3)	0.6833 (2)	0.62873 (14)	0.0430 (6)	
H28	0.7172	0.7642	0.6356	0.052*	
C29	0.8279 (2)	0.6086 (2)	0.58637 (13)	0.0391 (6)	
H29	0.8995	0.6381	0.5635	0.047*	
C30	0.8329 (2)	0.4914 (2)	0.57690 (12)	0.0307 (5)	
H30	0.9081	0.4403	0.5476	0.037*	
C31	0.8067 (2)	0.2133 (2)	0.71060 (11)	0.0277 (5)	
H31A	0.8220	0.1262	0.7206	0.033*	
H31B	0.7129	0.2517	0.7286	0.033*	
C32	0.8813 (2)	0.2607 (2)	0.75521 (12)	0.0300 (5)	
H32A	0.8832	0.2131	0.8010	0.036*	
H32B	0.9721	0.2464	0.7275	0.036*	
C33	0.8288 (2)	0.39255 (19)	0.77481 (12)	0.0282 (5)	
H33A	0.7343	0.4110	0.7956	0.034*	
H33B	0.8415	0.4418	0.7299	0.034*	
C34	0.8954 (2)	0.4262 (2)	0.82971 (12)	0.0307 (5)	
H34A	0.8868	0.3733	0.8732	0.037*	
H34B	0.9891	0.4110	0.8077	0.037*	
C35	0.8410 (2)	0.5560 (2)	0.85387 (12)	0.0329 (5)	
H35A	0.7462	0.5728	0.8731	0.040*	
H35B	0.8547	0.6090	0.8109	0.040*	
C36	0.9027 (2)	0.5862 (2)	0.91201 (13)	0.0339 (5)	
H36A	0.9970	0.5723	0.8919	0.041*	
H36B	0.8923	0.5307	0.9540	0.041*	
C37	0.8459 (2)	0.7147 (2)	0.93915 (13)	0.0323 (5)	
H37A	0.8583	0.7702	0.8974	0.039*	
H37B	0.7513	0.7292	0.9582	0.039*	
C38	0.9059 (2)	0.7432 (2)	0.99842 (12)	0.0318 (5)	
H38A	1.0005	0.7285	0.9793	0.038*	
H38B	0.8935	0.6876	1.0402	0.038*	
C39	0.8494 (2)	0.8716 (2)	1.02573 (12)	0.0308 (5)	
H39A	0.8610	0.9273	0.9839	0.037*	
H39B	0.7550	0.8861	1.0453	0.037*	
C40	0.9106 (2)	0.8998 (2)	1.08433 (12)	0.0314 (5)	
H40A	1.0051	0.8851	1.0647	0.038*	
H40B	0.8990	0.8441	1.1261	0.038*	
C41	0.8545 (2)	1.0287 (2)	1.11195 (12)	0.0323 (5)	
H41A	0.8642	1.0848	1.0702	0.039*	
H41B	0.7605	1.0430	1.1331	0.039*	
C42	0.9204 (2)	1.0552 (2)	1.16927 (13)	0.0391 (6)	
H42A	1.0146	1.0389	1.1481	0.047*	
H42B	0.9098	0.9991	1.2109	0.047*	
C43	0.8696 (3)	1.1830 (2)	1.19806 (14)	0.0480 (7)	
H43A	0.9298	1.1967	1.2263	0.058*	
H43B	0.8681	1.2401	1.1563	0.058*	
C44	0.7356 (3)	1.2077 (3)	1.24580 (16)	0.0590 (8)	
H44A	0.6753	1.1952	1.2179	0.071*	
H44B	0.7069	1.2909	1.2625	0.071*	
H44C	0.7370	1.1532	1.2881	0.071*	

### Atomic displacement parameters (Å^2^)

**Table:**

	*U*^11^	*U*^22^	*U*^33^	*U*^12^	*U*^13^	*U*^23^
Zn1	0.02638 (15)	0.02349 (14)	0.02330 (14)	−0.00954 (11)	−0.00226 (10)	−0.00692 (10)
S1	0.0280 (3)	0.0263 (3)	0.0206 (3)	−0.0076 (2)	−0.0048 (2)	−0.0019 (2)
S2	0.0276 (3)	0.0223 (3)	0.0270 (3)	−0.0070 (2)	−0.0050 (2)	0.0018 (2)
S3	0.0277 (3)	0.0216 (3)	0.0215 (3)	−0.0105 (2)	−0.0037 (2)	−0.0017 (2)
S4	0.0300 (3)	0.0246 (3)	0.0215 (3)	−0.0030 (2)	−0.0067 (2)	−0.0061 (2)
N1	0.0254 (9)	0.0223 (9)	0.0216 (9)	−0.0097 (7)	−0.0050 (7)	−0.0034 (7)
N2	0.0228 (9)	0.0213 (9)	0.0235 (9)	−0.0085 (7)	−0.0034 (7)	−0.0062 (7)
C1	0.0287 (11)	0.0158 (10)	0.0230 (11)	−0.0073 (9)	−0.0054 (9)	−0.0080 (8)
C2	0.0242 (11)	0.0310 (12)	0.0303 (12)	−0.0114 (10)	−0.0079 (9)	−0.0034 (9)
C3	0.0254 (11)	0.0305 (12)	0.0247 (11)	−0.0082 (10)	−0.0093 (9)	0.0020 (9)
C4	0.0266 (12)	0.0379 (14)	0.0404 (14)	−0.0101 (11)	−0.0070 (10)	−0.0013 (11)
C5	0.0271 (13)	0.0409 (15)	0.0493 (16)	0.0004 (11)	−0.0007 (11)	−0.0074 (12)
C6	0.0434 (15)	0.0258 (13)	0.0491 (16)	−0.0016 (11)	−0.0130 (12)	−0.0045 (11)
C7	0.0403 (14)	0.0267 (13)	0.0466 (15)	−0.0129 (11)	−0.0120 (12)	0.0043 (11)
C8	0.0261 (12)	0.0307 (13)	0.0323 (12)	−0.0081 (10)	−0.0052 (10)	0.0021 (10)
C9	0.0289 (12)	0.0267 (12)	0.0267 (11)	−0.0141 (10)	−0.0015 (9)	−0.0030 (9)
C10	0.0292 (12)	0.0316 (12)	0.0236 (11)	−0.0120 (10)	−0.0028 (9)	−0.0021 (9)
C11	0.0302 (12)	0.0316 (12)	0.0244 (11)	−0.0129 (10)	−0.0045 (9)	−0.0029 (9)
C12	0.0352 (13)	0.0404 (14)	0.0280 (12)	−0.0168 (11)	−0.0043 (10)	−0.0075 (10)
C13	0.0410 (13)	0.0371 (13)	0.0286 (12)	−0.0187 (11)	−0.0053 (10)	−0.0046 (10)
C14	0.0352 (13)	0.0374 (14)	0.0329 (13)	−0.0143 (11)	−0.0050 (10)	−0.0088 (10)
C15	0.0401 (13)	0.0346 (13)	0.0311 (12)	−0.0162 (11)	−0.0055 (10)	−0.0068 (10)
C16	0.0342 (13)	0.0349 (13)	0.0349 (13)	−0.0101 (11)	−0.0078 (10)	−0.0082 (10)
C17	0.0400 (13)	0.0344 (13)	0.0316 (13)	−0.0147 (11)	−0.0078 (10)	−0.0065 (10)
C18	0.0338 (13)	0.0341 (13)	0.0344 (13)	−0.0111 (11)	−0.0057 (10)	−0.0068 (10)
C19	0.0365 (13)	0.0328 (13)	0.0294 (12)	−0.0127 (11)	−0.0037 (10)	−0.0050 (10)
C20	0.0371 (13)	0.0309 (13)	0.0277 (12)	−0.0099 (10)	−0.0075 (10)	−0.0035 (10)
C21	0.0569 (16)	0.0363 (14)	0.0337 (13)	−0.0178 (12)	−0.0089 (12)	−0.0069 (11)
C22	0.081 (2)	0.0381 (15)	0.0388 (15)	−0.0133 (14)	−0.0196 (14)	−0.0083 (12)
C23	0.0257 (11)	0.0207 (10)	0.0218 (10)	−0.0142 (9)	−0.0037 (9)	−0.0037 (8)
C24	0.0210 (11)	0.0259 (12)	0.0325 (12)	−0.0051 (9)	−0.0066 (9)	−0.0079 (9)
C25	0.0257 (11)	0.0213 (11)	0.0200 (10)	−0.0048 (9)	−0.0077 (9)	−0.0024 (8)
C26	0.0271 (12)	0.0306 (13)	0.0343 (13)	−0.0045 (10)	−0.0033 (10)	−0.0058 (10)
C27	0.0452 (15)	0.0300 (13)	0.0455 (15)	0.0058 (12)	−0.0110 (12)	−0.0146 (11)
C28	0.0699 (18)	0.0204 (12)	0.0445 (15)	−0.0114 (13)	−0.0259 (14)	0.0016 (11)
C29	0.0547 (16)	0.0349 (14)	0.0349 (14)	−0.0250 (13)	−0.0107 (12)	0.0084 (11)
C30	0.0348 (13)	0.0302 (12)	0.0268 (12)	−0.0122 (10)	−0.0013 (10)	−0.0022 (9)
C31	0.0279 (12)	0.0294 (12)	0.0242 (11)	−0.0099 (10)	0.0018 (9)	−0.0051 (9)
C32	0.0307 (12)	0.0330 (13)	0.0252 (11)	−0.0063 (10)	−0.0042 (9)	−0.0093 (9)
C33	0.0317 (12)	0.0287 (12)	0.0257 (11)	−0.0091 (10)	−0.0069 (9)	−0.0045 (9)
C34	0.0355 (13)	0.0311 (13)	0.0264 (12)	−0.0074 (10)	−0.0089 (10)	−0.0062 (9)
C35	0.0378 (13)	0.0321 (13)	0.0304 (12)	−0.0076 (11)	−0.0115 (10)	−0.0050 (10)
C36	0.0365 (13)	0.0330 (13)	0.0340 (13)	−0.0081 (11)	−0.0105 (10)	−0.0099 (10)
C37	0.0352 (13)	0.0309 (13)	0.0331 (13)	−0.0090 (10)	−0.0111 (10)	−0.0045 (10)
C38	0.0351 (13)	0.0320 (13)	0.0308 (12)	−0.0099 (10)	−0.0091 (10)	−0.0068 (10)
C39	0.0358 (13)	0.0304 (12)	0.0278 (12)	−0.0103 (10)	−0.0078 (10)	−0.0030 (10)
C40	0.0343 (13)	0.0334 (13)	0.0277 (12)	−0.0099 (10)	−0.0064 (10)	−0.0059 (10)
C41	0.0392 (13)	0.0307 (13)	0.0279 (12)	−0.0114 (11)	−0.0061 (10)	−0.0025 (10)
C42	0.0410 (14)	0.0441 (15)	0.0349 (14)	−0.0134 (12)	−0.0074 (11)	−0.0130 (11)
C43	0.0604 (17)	0.0489 (16)	0.0404 (15)	−0.0254 (14)	−0.0051 (13)	−0.0131 (12)
C44	0.0670 (19)	0.0537 (18)	0.0540 (18)	−0.0136 (16)	−0.0067 (15)	−0.0190 (15)

### Geometric parameters (Å, °)

**Table:**

Zn1—S4	2.3396 (6)	C20—H20B	0.9900
Zn1—S2	2.3398 (6)	C21—C22	1.517 (3)
Zn1—S3	2.3711 (6)	C21—H21A	0.9900
Zn1—S1	2.4420 (6)	C21—H21B	0.9900
Zn1—S3^i^	2.8879 (6)	C22—H22A	0.9800
S1—C1	1.726 (2)	C22—H22B	0.9800
S2—C1	1.732 (2)	C22—H22C	0.9800
S3—C23	1.748 (2)	C23—S4^i^	1.720 (2)
S4—C23^i^	1.720 (2)	C24—C25	1.502 (3)
N1—C1	1.333 (3)	C24—H24A	0.9900
N1—C9	1.472 (3)	C24—H24B	0.9900
N1—C2	1.478 (3)	C25—C30	1.383 (3)
N2—C23	1.323 (2)	C25—C26	1.383 (3)
N2—C24	1.479 (3)	C26—C27	1.386 (3)
N2—C31	1.486 (3)	C26—H26	0.9500
C2—C3	1.514 (3)	C27—C28	1.368 (4)
C2—H2A	0.9900	C27—H27	0.9500
C2—H2B	0.9900	C28—C29	1.373 (3)
C3—C8	1.387 (3)	C28—H28	0.9500
C3—C4	1.390 (3)	C29—C30	1.373 (3)
C4—C5	1.386 (3)	C29—H29	0.9500
C4—H4	0.9500	C30—H30	0.9500
C5—C6	1.381 (3)	C31—C32	1.525 (3)
C5—H5	0.9500	C31—H31A	0.9900
C6—C7	1.376 (3)	C31—H31B	0.9900
C6—H6	0.9500	C32—C33	1.518 (3)
C7—C8	1.382 (3)	C32—H32A	0.9900
C7—H7	0.9500	C32—H32B	0.9900
C8—H8	0.9500	C33—C34	1.525 (3)
C9—C10	1.525 (3)	C33—H33A	0.9900
C9—H9A	0.9900	C33—H33B	0.9900
C9—H9B	0.9900	C34—C35	1.520 (3)
C10—C11	1.514 (3)	C34—H34A	0.9900
C10—H10A	0.9900	C34—H34B	0.9900
C10—H10B	0.9900	C35—C36	1.522 (3)
C11—C12	1.521 (3)	C35—H35A	0.9900
C11—H11A	0.9900	C35—H35B	0.9900
C11—H11B	0.9900	C36—C37	1.523 (3)
C12—C13	1.513 (3)	C36—H36A	0.9900
C12—H12A	0.9900	C36—H36B	0.9900
C12—H12B	0.9900	C37—C38	1.519 (3)
C13—C14	1.513 (3)	C37—H37A	0.9900
C13—H13A	0.9900	C37—H37B	0.9900
C13—H13B	0.9900	C38—C39	1.523 (3)
C14—C15	1.518 (3)	C38—H38A	0.9900
C14—H14A	0.9900	C38—H38B	0.9900
C14—H14B	0.9900	C39—C40	1.516 (3)
C15—C16	1.518 (3)	C39—H39A	0.9900
C15—H15A	0.9900	C39—H39B	0.9900
C15—H15B	0.9900	C40—C41	1.530 (3)
C16—C17	1.515 (3)	C40—H40A	0.9900
C16—H16A	0.9900	C40—H40B	0.9900
C16—H16B	0.9900	C41—C42	1.523 (3)
C17—C18	1.524 (3)	C41—H41A	0.9900
C17—H17A	0.9900	C41—H41B	0.9900
C17—H17B	0.9900	C42—C43	1.525 (3)
C18—C19	1.512 (3)	C42—H42A	0.9900
C18—H18A	0.9900	C42—H42B	0.9900
C18—H18B	0.9900	C43—C44	1.504 (4)
C19—C20	1.521 (3)	C43—H43A	0.9900
C19—H19A	0.9900	C43—H43B	0.9900
C19—H19B	0.9900	C44—H44A	0.9800
C20—C21	1.514 (3)	C44—H44B	0.9800
C20—H20A	0.9900	C44—H44C	0.9800
			
S4—Zn1—S2	136.18 (2)	C21—C22—H22A	109.5
S4—Zn1—S3	103.77 (2)	C21—C22—H22B	109.5
S2—Zn1—S3	114.12 (2)	H22A—C22—H22B	109.5
S4—Zn1—S1	108.81 (2)	C21—C22—H22C	109.5
S2—Zn1—S1	75.71 (2)	H22A—C22—H22C	109.5
S3—Zn1—S1	113.87 (2)	H22B—C22—H22C	109.5
S1—Zn1—S3^i^	154.92 (2)	N2—C23—S4^i^	120.54 (15)
C1—S1—Zn1	82.52 (7)	N2—C23—S3	121.74 (16)
C1—S2—Zn1	85.54 (7)	S4^i^—C23—S3	117.72 (11)
C23—S3—Zn1	97.24 (6)	N2—C24—C25	113.18 (17)
C23^i^—S4—Zn1	95.32 (7)	N2—C24—H24A	108.9
C1—N1—C9	121.94 (17)	C25—C24—H24A	108.9
C1—N1—C2	121.80 (17)	N2—C24—H24B	108.9
C9—N1—C2	115.75 (16)	C25—C24—H24B	108.9
C23—N2—C24	122.55 (17)	H24A—C24—H24B	107.8
C23—N2—C31	120.41 (17)	C30—C25—C26	118.7 (2)
C24—N2—C31	116.19 (16)	C30—C25—C24	120.33 (18)
N1—C1—S1	122.21 (16)	C26—C25—C24	121.01 (18)
N1—C1—S2	121.59 (16)	C25—C26—C27	120.3 (2)
S1—C1—S2	116.20 (11)	C25—C26—H26	119.9
N1—C2—C3	113.80 (17)	C27—C26—H26	119.9
N1—C2—H2A	108.8	C28—C27—C26	120.3 (2)
C3—C2—H2A	108.8	C28—C27—H27	119.9
N1—C2—H2B	108.8	C26—C27—H27	119.9
C3—C2—H2B	108.8	C27—C28—C29	119.8 (2)
H2A—C2—H2B	107.7	C27—C28—H28	120.1
C8—C3—C4	118.3 (2)	C29—C28—H28	120.1
C8—C3—C2	121.65 (18)	C28—C29—C30	120.3 (2)
C4—C3—C2	120.08 (19)	C28—C29—H29	119.9
C5—C4—C3	120.7 (2)	C30—C29—H29	119.9
C5—C4—H4	119.7	C29—C30—C25	120.8 (2)
C3—C4—H4	119.7	C29—C30—H30	119.6
C6—C5—C4	120.5 (2)	C25—C30—H30	119.6
C6—C5—H5	119.8	N2—C31—C32	117.10 (17)
C4—C5—H5	119.8	N2—C31—H31A	108.0
C7—C6—C5	119.1 (2)	C32—C31—H31A	108.0
C7—C6—H6	120.5	N2—C31—H31B	108.0
C5—C6—H6	120.5	C32—C31—H31B	108.0
C6—C7—C8	120.7 (2)	H31A—C31—H31B	107.3
C6—C7—H7	119.6	C33—C32—C31	116.30 (18)
C8—C7—H7	119.6	C33—C32—H32A	108.2
C7—C8—C3	120.8 (2)	C31—C32—H32A	108.2
C7—C8—H8	119.6	C33—C32—H32B	108.2
C3—C8—H8	119.6	C31—C32—H32B	108.2
N1—C9—C10	115.12 (17)	H32A—C32—H32B	107.4
N1—C9—H9A	108.5	C32—C33—C34	112.19 (18)
C10—C9—H9A	108.5	C32—C33—H33A	109.2
N1—C9—H9B	108.5	C34—C33—H33A	109.2
C10—C9—H9B	108.5	C32—C33—H33B	109.2
H9A—C9—H9B	107.5	C34—C33—H33B	109.2
C11—C10—C9	114.41 (17)	H33A—C33—H33B	107.9
C11—C10—H10A	108.7	C35—C34—C33	113.98 (18)
C9—C10—H10A	108.7	C35—C34—H34A	108.8
C11—C10—H10B	108.7	C33—C34—H34A	108.8
C9—C10—H10B	108.7	C35—C34—H34B	108.8
H10A—C10—H10B	107.6	C33—C34—H34B	108.8
C10—C11—C12	112.98 (17)	H34A—C34—H34B	107.7
C10—C11—H11A	109.0	C34—C35—C36	113.37 (19)
C12—C11—H11A	109.0	C34—C35—H35A	108.9
C10—C11—H11B	109.0	C36—C35—H35A	108.9
C12—C11—H11B	109.0	C34—C35—H35B	108.9
H11A—C11—H11B	107.8	C36—C35—H35B	108.9
C13—C12—C11	113.47 (18)	H35A—C35—H35B	107.7
C13—C12—H12A	108.9	C35—C36—C37	114.20 (19)
C11—C12—H12A	108.9	C35—C36—H36A	108.7
C13—C12—H12B	108.9	C37—C36—H36A	108.7
C11—C12—H12B	108.9	C35—C36—H36B	108.7
H12A—C12—H12B	107.7	C37—C36—H36B	108.7
C14—C13—C12	115.53 (18)	H36A—C36—H36B	107.6
C14—C13—H13A	108.4	C38—C37—C36	113.87 (19)
C12—C13—H13A	108.4	C38—C37—H37A	108.8
C14—C13—H13B	108.4	C36—C37—H37A	108.8
C12—C13—H13B	108.4	C38—C37—H37B	108.8
H13A—C13—H13B	107.5	C36—C37—H37B	108.8
C13—C14—C15	113.56 (18)	H37A—C37—H37B	107.7
C13—C14—H14A	108.9	C37—C38—C39	114.07 (19)
C15—C14—H14A	108.9	C37—C38—H38A	108.7
C13—C14—H14B	108.9	C39—C38—H38A	108.7
C15—C14—H14B	108.9	C37—C38—H38B	108.7
H14A—C14—H14B	107.7	C39—C38—H38B	108.7
C14—C15—C16	114.49 (18)	H38A—C38—H38B	107.6
C14—C15—H15A	108.6	C40—C39—C38	113.79 (19)
C16—C15—H15A	108.6	C40—C39—H39A	108.8
C14—C15—H15B	108.6	C38—C39—H39A	108.8
C16—C15—H15B	108.6	C40—C39—H39B	108.8
H15A—C15—H15B	107.6	C38—C39—H39B	108.8
C17—C16—C15	113.85 (18)	H39A—C39—H39B	107.7
C17—C16—H16A	108.8	C39—C40—C41	114.09 (19)
C15—C16—H16A	108.8	C39—C40—H40A	108.7
C17—C16—H16B	108.8	C41—C40—H40A	108.7
C15—C16—H16B	108.8	C39—C40—H40B	108.7
H16A—C16—H16B	107.7	C41—C40—H40B	108.7
C16—C17—C18	114.13 (19)	H40A—C40—H40B	107.6
C16—C17—H17A	108.7	C42—C41—C40	112.82 (19)
C18—C17—H17A	108.7	C42—C41—H41A	109.0
C16—C17—H17B	108.7	C40—C41—H41A	109.0
C18—C17—H17B	108.7	C42—C41—H41B	109.0
H17A—C17—H17B	107.6	C40—C41—H41B	109.0
C19—C18—C17	113.93 (18)	H41A—C41—H41B	107.8
C19—C18—H18A	108.8	C41—C42—C43	115.1 (2)
C17—C18—H18A	108.8	C41—C42—H42A	108.5
C19—C18—H18B	108.8	C43—C42—H42A	108.5
C17—C18—H18B	108.8	C41—C42—H42B	108.5
H18A—C18—H18B	107.7	C43—C42—H42B	108.5
C18—C19—C20	114.23 (18)	H42A—C42—H42B	107.5
C18—C19—H19A	108.7	C44—C43—C42	112.5 (2)
C20—C19—H19A	108.7	C44—C43—H43A	109.1
C18—C19—H19B	108.7	C42—C43—H43A	109.1
C20—C19—H19B	108.7	C44—C43—H43B	109.1
H19A—C19—H19B	107.6	C42—C43—H43B	109.1
C21—C20—C19	113.98 (19)	H43A—C43—H43B	107.8
C21—C20—H20A	108.8	C43—C44—H44A	109.5
C19—C20—H20A	108.8	C43—C44—H44B	109.5
C21—C20—H20B	108.8	H44A—C44—H44B	109.5
C19—C20—H20B	108.8	C43—C44—H44C	109.5
H20A—C20—H20B	107.7	H44A—C44—H44C	109.5
C20—C21—C22	113.7 (2)	H44B—C44—H44C	109.5
C20—C21—H21A	108.8	S4—Zn1—S3^i^	68.266 (19)
C22—C21—H21A	108.8	S2—Zn1—S3^i^	89.532 (19)
C20—C21—H21B	108.8	S3—Zn1—S3^i^	90.553 (19)
C22—C21—H21B	108.8	S1—Zn1—S3^i^	154.920 (19)
H21A—C21—H21B	107.7	C23^i^—S4—Zn1	95.32 (7)
			
S4—Zn1—S1—C1	133.25 (7)	C13—C14—C15—C16	179.7 (2)
S2—Zn1—S1—C1	−1.16 (6)	C14—C15—C16—C17	−178.5 (2)
S3—Zn1—S1—C1	−111.56 (7)	C15—C16—C17—C18	−178.2 (2)
S4—Zn1—S2—C1	−101.28 (7)	C16—C17—C18—C19	−178.9 (2)
S3—Zn1—S2—C1	111.24 (7)	C17—C18—C19—C20	−179.0 (2)
S1—Zn1—S2—C1	1.15 (6)	C18—C19—C20—C21	−179.9 (2)
S4—Zn1—S3—C23	−144.88 (7)	C19—C20—C21—C22	178.7 (2)
S2—Zn1—S3—C23	12.59 (7)	C24—N2—C23—S4^i^	−176.67 (14)
S1—Zn1—S3—C23	96.99 (7)	C31—N2—C23—S4^i^	−7.6 (2)
S2—Zn1—S4—C23^i^	−71.68 (7)	C24—N2—C23—S3	3.5 (3)
S3—Zn1—S4—C23^i^	77.98 (7)	C31—N2—C23—S3	172.55 (14)
S1—Zn1—S4—C23^i^	−160.44 (6)	Zn1—S3—C23—N2	−82.29 (16)
C9—N1—C1—S1	171.79 (14)	Zn1—S3—C23—S4^i^	97.90 (10)
C2—N1—C1—S1	0.3 (3)	C23—N2—C24—C25	−112.7 (2)
C9—N1—C1—S2	−7.7 (3)	C31—N2—C24—C25	77.9 (2)
C2—N1—C1—S2	−179.22 (14)	N2—C24—C25—C30	64.7 (3)
Zn1—S1—C1—N1	−177.87 (16)	N2—C24—C25—C26	−116.0 (2)
Zn1—S1—C1—S2	1.69 (9)	C30—C25—C26—C27	−0.9 (3)
Zn1—S2—C1—N1	177.81 (16)	C24—C25—C26—C27	179.8 (2)
Zn1—S2—C1—S1	−1.76 (10)	C25—C26—C27—C28	0.4 (4)
C1—N1—C2—C3	−98.4 (2)	C26—C27—C28—C29	0.4 (4)
C9—N1—C2—C3	89.6 (2)	C27—C28—C29—C30	−0.7 (4)
N1—C2—C3—C8	64.1 (3)	C28—C29—C30—C25	0.2 (4)
N1—C2—C3—C4	−116.5 (2)	C26—C25—C30—C29	0.6 (3)
C8—C3—C4—C5	−0.6 (3)	C24—C25—C30—C29	179.9 (2)
C2—C3—C4—C5	179.9 (2)	C23—N2—C31—C32	80.0 (2)
C3—C4—C5—C6	0.8 (4)	C24—N2—C31—C32	−110.3 (2)
C4—C5—C6—C7	−0.8 (4)	N2—C31—C32—C33	83.7 (2)
C5—C6—C7—C8	0.6 (4)	C31—C32—C33—C34	170.34 (18)
C6—C7—C8—C3	−0.4 (4)	C32—C33—C34—C35	−177.04 (18)
C4—C3—C8—C7	0.4 (3)	C33—C34—C35—C36	176.50 (19)
C2—C3—C8—C7	179.9 (2)	C34—C35—C36—C37	−177.71 (19)
C1—N1—C9—C10	121.0 (2)	C35—C36—C37—C38	178.65 (19)
C2—N1—C9—C10	−67.1 (2)	C36—C37—C38—C39	179.98 (19)
N1—C9—C10—C11	−56.0 (3)	C37—C38—C39—C40	−179.42 (19)
C9—C10—C11—C12	−177.38 (19)	C38—C39—C40—C41	179.92 (18)
C10—C11—C12—C13	−174.42 (19)	C39—C40—C41—C42	−178.47 (19)
C11—C12—C13—C14	−177.7 (2)	C40—C41—C42—C43	179.18 (19)
C12—C13—C14—C15	−179.3 (2)	C41—C42—C43—C44	70.9 (3)

Symmetry codes: (i) −*x*+2, −*y*, −*z*+1.
